# Automated Prediction of Neoadjuvant Chemoradiotherapy Response in Locally Advanced Cervical Cancer Using Hybrid Model-Based MRI Radiomics

**DOI:** 10.3390/diagnostics14010005

**Published:** 2023-12-19

**Authors:** Hua Yang, Yinan Xu, Mohan Dong, Ying Zhang, Jie Gong, Dong Huang, Junhua He, Lichun Wei, Shigao Huang, Lina Zhao

**Affiliations:** 1 Department of Radiation Oncology, Xijing Hospital of Air Force Medical University, Xi’an 710032, China; yanghuafmmu@163.com (H.Y.); zyingxa@gmail.com (Y.Z.); 18700182707@163.com (J.G.); 2 Department of Radiation Oncology, The First Affiliated Hospital of Xi’an Jiaotong University, Xi’an 710061, China; 3 Key Lab of Intelligent Perception and Image Understanding of Ministry of Education, Xidian University, Xi’an 710071, China; xyn1998102@163.com; 4 Department of Medical Education, Xijing Hospital of Air Force Medical University, Xi’an 710032, China; dongmohandd@163.com; 5 Department of Military Biomedical Engineering, Air Force Medical University, Xi’an 710012, China; huangdong1007785@outlook.com; 6 Department of Radiation Oncology, 986 Hospital of Air Force Medical University, Xi’an 710054, China; HJP20231216@163.com

**Keywords:** automated prediction, MRI radiomics, radiotherapy response, cervical cancer

## Abstract

Background: This study aimed to develop a model that automatically predicts the neoadjuvant chemoradiotherapy (nCRT) response for patients with locally advanced cervical cancer (LACC) based on T2-weighted MR images and clinical parameters. Methods: A total of 138 patients were enrolled, and T2-weighted MR images and clinical information of the patients before treatment were collected. Clinical information included age, stage, pathological type, squamous cell carcinoma (SCC) level, and lymph node status. A hybrid model extracted the domain-specific features from the computational radiomics system, the abstract features from the deep learning network, and the clinical parameters. Then, it employed an ensemble learning classifier weighted by logistic regression (LR) classifier, support vector machine (SVM) classifier, K-Nearest Neighbor (KNN) classifier, and Bayesian classifier to predict the pathologic complete response (pCR). The area under the receiver operating characteristics curve (AUC), accuracy (ACC), true positive rate (TPR), true negative rate (TNR), and precision were used as evaluation metrics. Results: Among the 138 LACC patients, 74 were in the pCR group, and 64 were in the non-pCR group. There was no significant difference between the two cohorts in terms of tumor diameter (*p* = 0.787), lymph node (*p* = 0.068), and stage before radiotherapy (*p* = 0.846), respectively. The 109-dimension domain features and 1472-dimension abstract features from MRI images were used to form a hybrid model. The average AUC, ACC, TPR, TNR, and precision of the proposed hybrid model were about 0.80, 0.71, 0.75, 0.66, and 0.71, while the AUC values of using clinical parameters, domain-specific features, and abstract features alone were 0.61, 0.67 and 0.76, respectively. The AUC value of the model without an ensemble learning classifier was 0.76. Conclusions: The proposed hybrid model can predict the radiotherapy response of patients with LACC, which might help radiation oncologists create personalized treatment plans for patients.

## 1. Introduction

According to statistics, there were nearly 110,000 newly diagnosed cervical cancer patients and nearly 60,000 deaths in China in 2020, accounting for 18.3% and 17.6% of the global total, respectively [1]. Radiotherapy is one of the main treatment options for cervical cancer. A 5-year overall survival rate of 70% can be achieved for locally advanced cervical cancer treated with 3D conformal intensity-modulated radiotherapy combined with brachytherapy [2,3]. However, some patients continue to have residual lesions after radiotherapy or experience recurrence or metastasis shortly after treatment, as well as face limited treatment options and poor prognosis [4,5,6,7,8,9,10]. This may be related to the sensitivity of tumors to radiotherapy. Therefore, predicting the radiotherapy sensitivity of patients with locally advanced cervical cancer before treatment may help doctors formulate more effective treatment strategies, especially for patients who are insensitive to radiotherapy.

Magnetic resonance imaging (MRI) is commonly used to accurately evaluate the extent of cervical cancer and treatment response [11]. Radiomics research extracts quantitative features from medical images using high-throughput techniques and has been increasingly used in tumor prognosis prediction in recent years [12,13,14,15,16]. There are currently two main ways to establish a predictive model using radiomics. One is to extract the physically defined features of the image, such as shape, size, and texture. The other is to use deep learning to extract abstract features for modeling. There have been reports of using radiomics features based on MRI to predict the long-term therapeutic effect of locally advanced cervical cancer after radiotherapy [17,18]. However, long-term therapeutic effects are influenced by many factors and may not be an ideal representation of radiotherapy sensitivity. The pathological complete response (pCR) is a commonly used indicator for evaluating the sensitivity of radiotherapy [13,14]. An Italian study used pCR as a predictive indicator for cervical cancer radiotherapy sensitivity, but this study mainly used specifically extracted T2-weighted image domain-specific features from MRI and inputted them into a random forest classifier for prediction [19]. However, this traditional radiomics only extracts domain-specific features from MRI. 

In this study, we also used pCR as a label to accurately reflect the neoadjuvant chemoradiotherapy (nCRT) response. By extracting multi-modal radiomics information features and using deep learning to extract abstract features, combined with clinical information features, we established a hybrid model for predicting the cervical cancer nCRT response for the first time. This model combines the advantages of computing domain-specific features of the radiomics system and deep learning-based abstract features, providing a new option for early prediction of the cervical cancer nCRT response.

## 2. Materials and Methods

### 2.1. Materials

This study was conducted in accordance with the Declaration of Helsinki and approved by the Ethics Committee of Xijing Hospital, the Air Force Medical University (KY20192025-F-1). The dataset used in our study was collected from the Department of Radiology, Xijing Hospital, Xi’an, China. A total of 138 cervical cancer patients from January 2015 to December 2019 were enrolled. For each patient, the MR image was acquired using a 3 T MR scanner (TrioTim, Siemens, Erlangen, Germany), and the repetition time/echo time (TR/TE) parameters of the T2-weighted Sequence was 5750/95 msec. The regions of interest for cervical cancer were contoured by the collaboration of experienced radiologists and medical professionals specialized in oncology and gynecology. The in-plane resolution on these images ranges from 0.5 mm to 1.25 mm, and the inter-slice thickness is 6 mm. 

**Figure 1:**
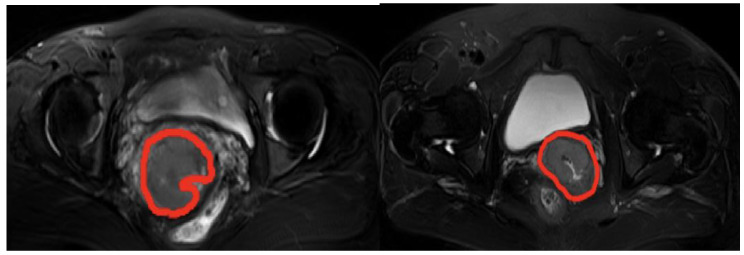
MRI images of a patient with pCR before radiotherapy (**left**) and a patient with non-pCR before radiotherapy (**right**). We used T2WI-FS here to provide a clearer image of the collected data; The red circles represent the tumor area.

On the other hand, several clinical parameters, including age, stage, pathological type, SCC level, and lymph node status, were used in this study. According to the postoperative pathological report, the patients were divided into two groups: pathologically complete response (pCR) and non-pathologically complete response (non-pCR) with any persistent tumor microscopically or macroscopically, which was used as the label to predict the prognosis of CC. The MRI images labeled with pCR and non-pCR are shown in Figure 1, and the clinical parameters are described in Table 1. 

The external beam radiotherapy dose was 45–50 Gy/25 fractions for the pelvis and 62.5 Gy/25 fractions for metastatic lymph nodes and was delivered using the volumetric modulated arc therapy or three-dimensional conformal radiotherapy technique. All patients received concurrent chemotherapy with cisplatin 40 mg/m^2^ weekly. All patients underwent radical hysterectomy plus pelvic lymphadenectomy within 3–6 weeks of nCRT completion.

### 2.2. Methods

#### 2.2.1. Methodological Framework

**Figure 2:**
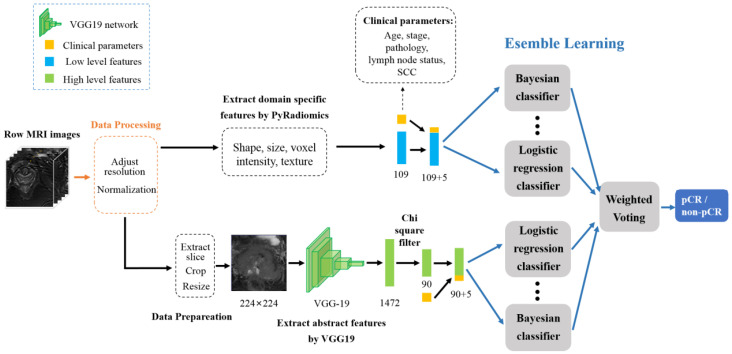
The pipeline of the proposed method.

The hybrid model consists of three stages: preprocessing, feature extraction, and pCR prediction. The domain-specific abstract and clinical parameter features were used to construct two feature vectors. The two feature vectors were, respectively, input into an ensemble learning classifier to obtain the final prediction results. The pipeline of the method is shown in Figure 2.

#### 2.2.2. Data Preprocessing

In this study, all MRI images were interpolated using the Simple ITK toolkit [20,21], and the resolution was uniformly interpolated to 0.5 mm × 0.5 mm × 3 mm. Moreover, the images were normalized to the range of [0–1].

#### 2.2.3. Extraction of Domain-Specific Features

The domain-specific features were extracted using a PyRadiomics open-source package [22]. Shape-based features describe the shape characteristics of gross tumor volume (GTV) contour, and the first-order statistics represent the distribution of voxel intensity in the GTV. These features based on gray-level matrices contain the size and texture of the GTV. Moreover, the clinical parameters were concatenated with the domain-specific features to form a domain feature vector for pCR prediction.

#### 2.2.4. Extraction of Abstract Features

The abstract features were extracted using a deep learning network and were selected by implementing the chi-square test. The selected features were directly concatenated to clinical parameters to form an abstract feature vector for pCR prediction.

Notably, we first constructed a VGG19 deep network. Then, the ImageNet dataset, which contains 1.2 million images, was used to train this VGG19 network. Based on transfer learning, the pre-trained VGG19 network was fine-tuned using our MRI images so that the abstract features of MRI images could be extracted from the fine-tuned VGG19 network. Specifically, for each patient’s image, the slice with the largest region of interest (ROI) was selected. These images were cropped to contain only the tumor area. To match the input of the fine-tuned VGG19 network, the images were extrapolated to a size of 224×224, and the grayscale images were converted into three-channel images by channel copying. Then, the images were fed into the fine-tuned VGG19 network to extract features. 

**Figure 3:**
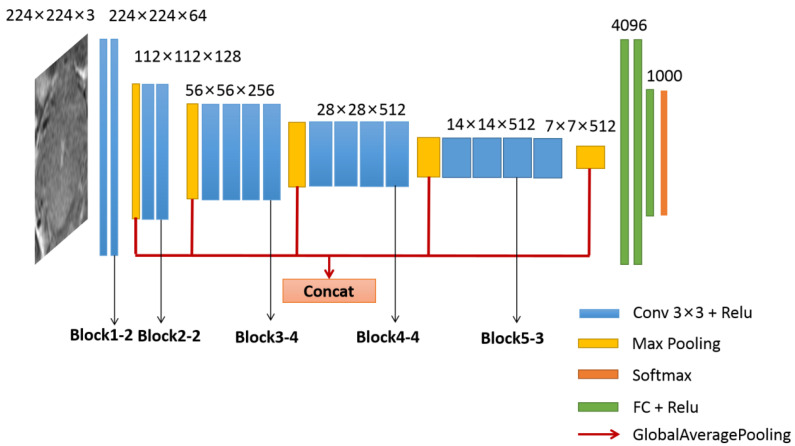
The structure of the VGG19 network.

In addition, the method proposed by Antropova et al. [23] was adopted. As shown in Figure 3, five feature vectors were obtained by adding a global average pooling layer (red line) after each max pooling layer (yellow block). Further, a feature vector consisting of 1472 dimensions was obtained by connecting these 5 feature vectors. To reduce feature dimension, the chi-square test feature-selection method was used.

#### 2.2.5. Prediction of pCR-Based on Ensemble Learning

**Figure 4:**
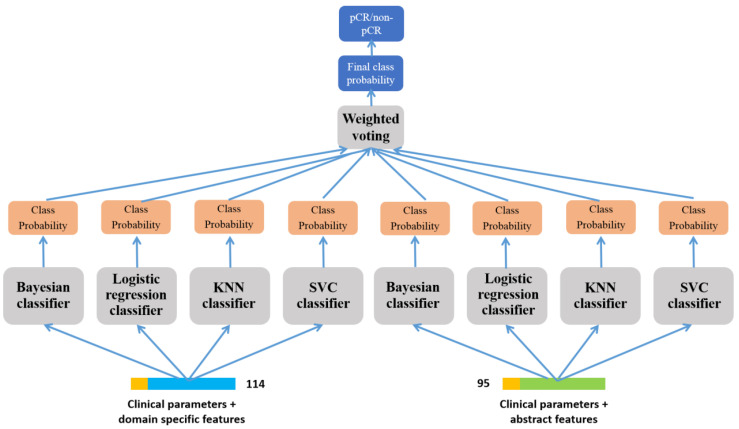
Ensemble learning flow charts for pCR/non-pCR prediction.

To overcome the limitation wherein a single classifier cannot perform best, we employed ensemble learning, which can aggregate the findings of numerous classifiers to produce a better and more stable classifier. As shown in Figure 4, the specific ensemble method is as follows. The two feature vectors introduced in the previous two sections were inputted into the LR classifier, Bayesian classifier, KNN classifier, and SVM classifier, respectively, and multiple class probability vectors were obtained. Further, an adaptive weighted voting strategy was used to vote on the class probability vectors from these classifiers to obtain the final class probability vectors. The weights were generated based on the performance of each classifier, which means that classifiers with higher accuracy for the validation data have greater weights. The prediction result of pCR was the class with the highest probability in the final class probability vectors, i.e., pCR or non-pCR.

Formally, the final prediction class *H(x)* is

(1) $$H(x)=\underset{j}{\arg\max}\sum_{i=1}^{8}w_{i}h_{i}^{j}(x)$$

where $h_{i}^{j}(x)$ is the *j*-th class probability in the class probability vector of the *i*-th classifier, and the weight $w_{i}$ denotes the weight of the *i*-th classifier:

(2) $$w_{i}=\frac{\exp(x_{i})}{\sum_{j=1}^{8}\exp(x_{j})},i=1\ldots 8$$

(3) $$x_{i}=\log\frac{p_{i}}{1-p_{i}},i=1\ldots 8$$

where $p_{i}$ is the accuracy of the validation data of the *i*-th classifier. 

#### 2.2.6. Evaluation Metrics

To evaluate the proposed method, the evaluation metrics *ACC*, *TPR*, *TNR,* precision, and AUC value were used. AUC is the most relevant evaluation metric. The metrics are defined as follows:

(4) $$TPR=\frac{TP}{TP+FN}$$

(5) $$TNR=\frac{TN}{FP+TN}$$

(6) $$ACC=\frac{TP+TN}{TP+FN+FP+TN}$$

(7) $$precision=\frac{TP}{FP+TP}$$

where *TP* represents true positive, *TN* represents true negative, *FN* represents false negative, and *FP* represents false positive. The AUC value is the area under the receiver operating characteristic (ROC) curve. Moreover, these metrics are expected to be as large as possible. 

#### 2.2.7. Training and Validation

In the experimental setup, we employed a five-fold cross-validation procedure, which, in our case, was repeated 20 times, along with measurements of mean and standard deviation. It is important to note that the purpose of repeating the cross-validation was to obtain a more stable estimation of model performance. All experiments were conducted using Python and the sklearn open-source packages to ensure consistency and replicability in our methodologies. This approach aimed to provide a more comprehensive understanding of the model’s generalization capability.

## 3. Results

### 3.1. Clinical Characteristics of the Patients

Among 138 LACC patients, 74 were in the pCR group, and 64 were in the non-pCR group. The characteristics of all patients in the pCR and non-pCR groups are shown in Table 1. There was no significant difference between the two cohorts regarding tumor diameter, lymph node, and stage before radiotherapy, *p* = 0.787, 0.068, and 0.846, respectively. Also, no significant difference was observed in other terms between the two groups.

### 3.2. The Features Extracted from MRI and Feature Vectors

First, 109-dimension domain-specific features were extracted. The features were divided into four types, including shape, size, voxel intensity, and texture. Details on this domain-specific feature are shown in the Supplementary File. Moreover, the clinical parameters were connected with the 109 features to form a 114-dimensional domain feature vector for pCR prediction.

**Figure 5:**
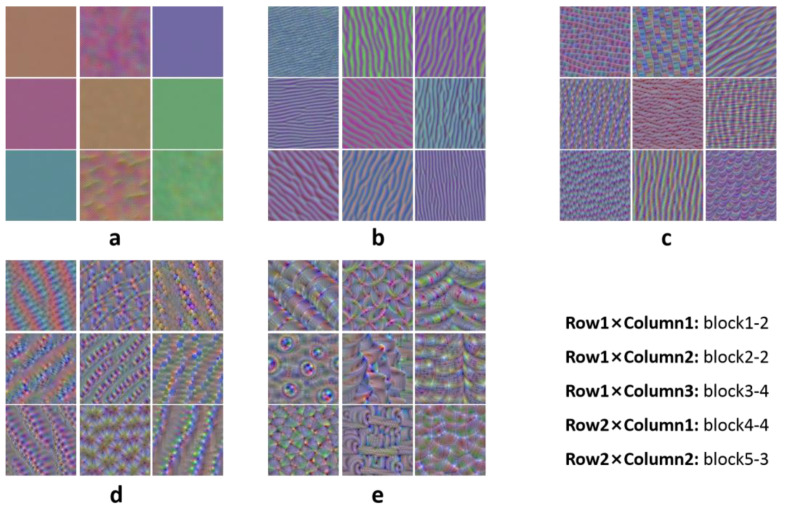
Maximum response graphs of convolution kernel in (**a**) Block1-2, (**b**) Block2-2, (**c**) Block3-4, (**d**) Block4-4, and (**e**) Block5-3 convolution layers of VGG19 network.

A total of 1472 dimension abstract features were extracted from the VGG19 network. The features from the VGG19 network were extracted at different levels; the maximum response graphs of some convolution kernels in the VGG19 network are shown in Figure 5. The low convolutional layers usually extract features such as color and edge (see Figure 5a,b), the middle convolutional layers extract texture features (see Figure 5c,d), and the high convolutional layers extract more structured and specific features of the classified objects (see Figure 5e). Finally, to form the feature vector, 90-dimension abstract features were selected from 1472-dimension features using the chi-square test. The selected features were directly concatenated to clinical parameters to form a 95-dimension abstract feature vector for pCR prediction.

### 3.3. Comparisons of the Different Features of Prediction Performance

To demonstrate the importance of using clinical parameters, domain-specific features, and abstract features together for pCR prediction, individual features and their combinations with different patterns were used to predict pCR, respectively.

**Figure 6:**
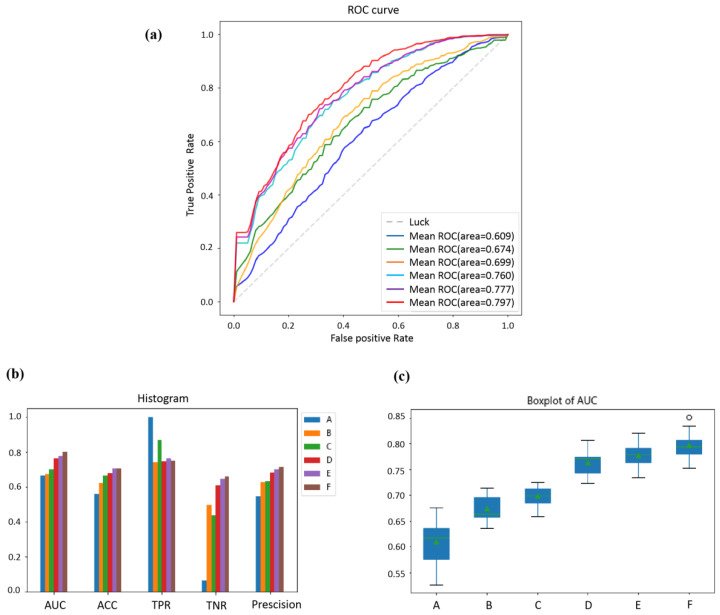
(**a**): The average ROC curves of the 20-times repeated five-fold cross-validation experiments of several classifiers trained by different features. (**b**): The histogram of AUC, ACC, TPR, and TNR, and the precision of the different classifiers. (**c**): Boxplot of the average AUC values of the classifiers constructed with the different features. A represents the classifier trained by clinical parameters; B represents the classifier trained by domain-specific features; C represents the classifier trained by clinical parameters and domain-specific features; D represents the classifier trained by abstract features; E represents the classifier trained by abstract features and clinical parameters; and F represents the classifier trained by abstract features, domain-specific features, and clinical parameters.

As the concerned evaluation metric, the AUC values and ROC curves of classifiers trained by different features are shown in Figure 6a. The classifier trained only by clinical parameters is the worst. Moreover, the domain-specific features of MRI images can effectively predict pCR. The clinical parameters can improve the classifier’s performance further. The classifier trained by abstract features is better than that trained by domain-specific features, which indicates that the abstract features extracted by transfer learning are more effective. The combination of abstract features and clinical parameters can achieve better performance. In addition, after ensemble learning is used, almost all metrics have been further improved, which shows that the single type of feature is not the best solution.

More metrics were used to compare the effects of different feature combinations on prediction performance, and a histogram was drawn to visualize the differences in Figure 6b. The detailed quantitative evaluation results of AUC, ACC, TPR, TNR, and precision are listed in Table 2. We compared six experimental groups—including single-feature, two-feature combinations, and three-feature combinations—to ascertain which combination would yield the best results. Given the results, it is evident that the combination of clinical parameters, domain-specific features, and abstract features (denoted as F) consistently demonstrated superior performance across most metrics. Notably, classifier F exhibited the highest AUC value of 0.797 ± 0.023, indicating strong discriminatory capability. 

To compare the influence of different features on the stability of the classifiers, the boxplot of AUC of 20 times experiments is shown in Figure 6c. As can be seen, the proposed method integrating all three types of features is more stable.

### 3.4. Comparisons of the Effect of Different Classifiers on Prediction Performance

Given the experimental results mentioned in the previous section, combining clinical parameters, domain-specific features, and abstract features achieves the best performance. However, how to organize them to build a better classifier is also a key issue. To this end, we compared the difference between the proposed ensemble classifier and the single classifier, including the Bayesian classifier, logistic regression classifier, KNN classifier, and SVC classifier. The input of a single classifier is the feature vector obtained by concatenating these three features.

In Table 3, compared to the proposed method shown in the fifth line, an unsatisfactory performance is shown for a single classifier on the first four lines. The reason for this result might be that the vector concatenation by three-type features has a high dimension, but the number of samples is insufficient. A solution to this is to feature selection combined with an ensemble learning classifier—all metrics are improved with this method. 

## 4. Discussion

In this paper, we developed a hybrid model that could automatically predict treatment responses for patients with LACC before neoadjuvant chemoradiotherapy (nCRT). The model is based on three types of features (domain features, abstract features, and clinical parameters) and the ensemble learning classifier can forecast the tumor pCR of patients with cervical cancer treated with chemoradiotherapy. Further, the prediction performance of three kinds of features with an ensemble learning classifier is better than a single classifier, single feature, or simple combined features.

Studies have shown that cervical cancer (CC) has a slightly high signal or high signal on T2WI images and is separated from surrounding tissues, so it has a high value in MRI radiomics [24,25,26]. At present, there have been many studies to predict the recurrence and metastasis of cervical cancer after radiotherapy and chemotherapy according to the radiological characteristics. Altazi et al. extracted six types of dome-specific features from MR and trained a multivariable logistic regression model to better predict the efficacy of distant metastasis and local recurrence [18]. Ayushi et al. revealed an incremental role of radiomics in functional MR imaging that deploys ADC values for predicting recurrence and distant metastasis [27]. Zhou et al. reported that the T2WI-FS imaging label has high efficacy in evaluating patients with lymphatic metastasis and LVSI [28]. However, few models have been established to predict the response of nCRT for CC, especially with the gold standard pathological results as the label. 

Firstly, in our study, domain-specific features and abstract features were extracted from MRI images in CC patients, and these features were used to predict patients’ response to radiotherapy (i.e., pCR or non-pCR). These features from images were more effective for the prediction model than the clinical parameters-based model.

Secondly, a recent study extracted 1889 domain-specific features from T2-weighted MR [19], and the random forest classifier was used to create a radiomics model for LACC to predict the pathological complete response (pCR) after nCRT. The results showed that radiomics appeared to be a reliable tool in pCR prediction. In contrast, the domain-specific features and the abstract features from deep networks were used for prediction in our study, which resulted in a richer feature representation of LACC and better prediction results.

Lastly, regarding domain features, they have clear physical meaning but are hand-crafted. Comparatively, the features extracted by the deep transfer learning network are more abstract. Fortunately, there is a visible method that can be used to understand what features from the VGG19 network are extracted at different levels; the maximum response graphs of some convolution kernels in the VGG19 network are shown in Figure 5. As stated in the convolutional neural network, the low convolutional layers usually extract features such as color and edges, and the middle and high convolutional layers represent texture features and contain more structured and specific features of the classified objects. It is worth noting that the feature-extraction method used in our study can fully use the features of all levels, unlike other studies [29,30], which only involved high-level features.

Also, deep learning plays an important role in the diagnosis and efficacy prediction of treatment outcomes in multiple tumors, such as urinary tumors, lung cancer, and brain tumors [31,32,33,34]. The changes in genes and history are important mechanisms of tumor occurrence and treatment resistance. Many studies have focused on judging gene changes and treatment responses of tumors using deep learning methods. Shi-r used a deep artificial neural network to predict the gene status of KRAS, NRAS, and BRAF in patients with liver metastasis of colorectal cancer [35]. Smedley-NF also used deep neural networks and interpretation methods to integrate gene and imaging features in non-small cell lung cancer to understand tumor heterogeneity, tumor tissue characteristics, and response to treatment [33]. In the future, deep learning will be combined with radiomics and genomics to represent the molecular and histological characteristics of cervical cancer and aid in implementing an accurate treatment.

However, the limitations of this study are as follows: 1. Given the challenge of obtaining pathological tumor results, our dataset comprises samples from 138 patients. While the dataset size is limited, we employed a stringent five-fold cross-validation approach to prevent data leakage. Furthermore, to ensure the model’s generalizability, we meticulously trained the model from scratch and performed 20 repeated experiments, randomly partitioning the data each time. In the future, expanding our sample size is crucial to rigorously validate the efficacy of our proposed model. 2. This is a retrospective study; the conventional pelvic MRI did not include diffusion sequence then, so we only extracted the features from the T2WI sequence. More sequence features can be combined, such as the dispersion sequence, which may extract more representative features. 

## 5. Conclusions

In conclusion, the proposed hybrid model based on three types of features and the ensemble learning classifier can accurately predict the treatment response of patients with cervical cancer. Using this hybrid model can help radiation oncologists in their decision-making process and in designing personalized treatment plans for patients with LACC.

## Figures and Tables

**Table 1:** Characteristics of the patients in pCR and non-pCR cohorts (χ^2^ test).

Parameters	pCR Group n (%) n = 74	Non-pCR Group n (%) n = 64	*p* Value
Age (years)			
median(range)	48 (25–64)	47 (27–65)	0.401
Tumor diameter (cm)			0.787
<4	20 (27.0)	16 (25.0)	
≥4	54 (73.0)	48 (75.0)	
Lymph node			0.068
positive	24 (32.4)	12 (18.8)	
negative	50 (67.2)	52 (81.2)	
2018 FIGO stage			0.846
IB	3 (4.1)	4 (6.3)	
IIA-IIB	39 (52.7)	32 (50)	
IIIA-IIIC1	32 (43.2)	28 (43.8)	
Radiotherapy technology			0.886
3DRT	54 (73.0)	46 (71.9)	
IMRT	20 (27.0)	18 (28.1)	
Pathological type			0.116
Squamous carcinoma	73 (98.6)	59 (92.2)	
Adenocarcinoma	1 (1.4)	4 (6.3)	
Other	0	1 (1.6)	
SCC			0.117
<1.5	25 (33.8)	13 (20.3)	
1.5–5	16 (21.6)	12 (18.8)	
>5	11 (14.9)	19 (29.7)	
Unclear	22 (29.7)	20 (31.2)	
OTT (days)			0.707
Median (range)	35 (30–46)	34 (30–42)	

3DRT: three-dimensional conformal radiotherapy; IMRT: intensity-modulated radiation therapy; SCC: squamous cell carcinoma; OTT: over treatment time.

**Table 2:** Quantitative evaluation results of the classifiers trained by different features, where A, B, C, D, E, and F have the same meanings as those in Figure 6.

Classifier	AUC	ACC	TPR	TNR	Precision
A	0.609 ± 0.037	0.582 ± 0.029	0.649 ± 0.047	0.517 ± 0.053	0.599 ± 0.028
B	0.674 ± 0.023	0.622 ± 0.027	0.739 ± 0.034	0.494 ± 0.033	0.625 ± 0.026
C	0.699 ± 0.019	0.663 ± 0.019	0.866 ± 0.024	0.435 ± 0.032	0.633 ± 0.017
D	0.760 ± 0.022	0.678 ± 0.024	0.765 ± 0.031	0.611 ± 0.026	0.683 ± 0.022
E	0.777 ± 0.021	0.704 ± 0.019	0.763 ± 0.033	0.645 ± 0.032	0.702 ± 0.021
F	0.797 ± 0.023	0.705 ± 0.018	0.750 ± 0.026	0.660 ± 0.025	0.711 ± 0.020

**Table 3:** Quantitative evaluation results of different classification methods.

Classification Method	AUC	ACC	TPR	TNR	Precision
Bayesian	0.688 ± 0.041	0.620 ± 0.015	0.674 ± 0.015	0.594 ± 0.052	0.634 ± 0.024
Logistic Regression	0.757 ± 0.021	0.687 ± 0.021	0.749 ± 0.025	0.625 ± 0.031	0.694 ± 0.018
KNN	0.748 ± 0.017	0.691 ± 0.035	0.740 ± 0.030	0.650 ± 0.049	0.683 ± 0.027
SVC	0.732 ± 0.015	0.669 ± 0.047	0.715 ± 0.066	0.602 ± 0.019	0.687 ± 0.026
Ensemble classifier (ours)	0.797 ± 0.023	0.705 ± 0.018	0.750 ± 0.026	0.660 ± 0.025	0.711 ± 0.020

## Data Availability

Data are contained within the article.

## Supplementary Materials

The following are available online at https://www.mdpi.com/article/10.3390/diagnostics14010005/s1, Supplementary File.
