# Supplementary material for: Automated Prediction of Neoadjuvant Chemoradiotherapy Response in Locally Advanced Cervical Cancer Using Hybrid Model-Based MRI Radiomics
Source: Diagnostics (Basel). 2023 Dec 19;14(1):5. doi: 10.3390/diagnostics14010005 (PMC10795804; doi:10.3390/diagnostics14010005)
Supplement: Supplementary file 1 [file diagnostics-14-00005-s001.zip › diagnostics-2644083-supplementary.pdf]

109-dimension domain specific features were extracted. The features were divided into four types, including shape, size, voxel intensity, texture. The detail of this domain specific feature is shown in following table.

Type	Number	Feature
shape	1	original_shape_Flatness
	2	original_shape_Maximum2DDiameterRow
	3	original_shape_MajorAxisLength
	4	original_shape_Maximum2DDiameterColumn
size	5	original_glszm_LargeAreaEmphasis
	6	original_glszm_SizeZoneNonUniformity
	7	original_glszm_SmallAreaEmphasis
	8	original_glszm_LargeAreaHighGrayLevelEmphasis
	9	original_glszm_ZonePercentage
	10	original_glszm_SmallAreaLowGrayLevelEmphasis
voxel intensity	11	square_firstorder_Variance
	12	exponential_firstorder_Kurtosis
	13	wavelet-HHH_firstorder_Uniformity
	14	wavelet-LHH_firstorder_90Percentile
	15	wavelet-HLL_glszm_GrayLevelVariance
	16	wavelet-HHH_firstorder_Energy
	17	wavelet-HLH_firstorder_RobustMeanAbsoluteDeviation
	18	wavelet-LHH_firstorder_MeanAbsoluteDeviation
	19	wavelet-HLH_firstorder_Minimum
	20	wavelet-HHH_firstorder_TotalEnergy
	21	wavelet-HLL_firstorder_90Percentile
	22	wavelet-LHL_firstorder_TotalEnergy
	23	wavelet-HHH_firstorder_Variance
	24	wavelet-HLH_firstorder_InterquartileRange
	25	wavelet-LHH_firstorder_Variance
	26	exponential_firstorder_MeanAbsoluteDeviation
	27	wavelet-HHH_firstorder_Skewness
	28	wavelet-LHL_firstorder_Energy
	29	wavelet-HLH_firstorder_Energy
	30	squareroot_firstorder_Variance
	31	lbp-3D-k_firstorder_Minimum
	32	lbp-3D-k_firstorder_90Percentile
	33	lbp-3D-k_firstorder_Mean
	34	wavelet-LLH_firstorder_Skewness
	35	wavelet-LLL_firstorder_Skewness

	36	wavelet-HHL_firstorder_Variance
	37	wavelet-HHL_firstorder_Skewness
	38	wavelet-HLL_firstorder_90Percentile
	39	lbp-3D-m2_firstorder_MeanAbsoluteDeviation
	40	lbp-3D-m1_firstorder_90Percentile
texture	41	original_glcm_JointEntropy
	42	wavelet-LLH_glcm_Correlation
	43	original_ngtdm_Strength
	44	wavelet-LLH_glszm_SmallAreaLowGrayLevelEmphasis
	45	wavelet-HLH_glszm_SizeZoneNonUniformity
	46	wavelet-HHH_glcm_Imc1
	47	original_gldm_DependenceEntropy
	48	wavelet-LLH_glcm_ClusterTendency
	49	wavelet-LHL_glszm_GrayLevelNonUniformityNormalized
	50	wavelet-LLL_glszm_HighGrayLevelZoneEmphasis
	51	wavelet-LLL_ngtdm_Strength
	52	wavelet-HHH_glcm_SumSquares
	53	wavelet-HLL_glrlm_RunPercentage
	54	wavelet-LLH_glcm_Idmn
	55	wavelet-HLL_glszm_GrayLevelNonUniformityNormalized
	56	wavelet-LHL_glrlm_GrayLevelVariance
	57	wavelet-LLH_glcm_DifferenceEntropy
	58	wavelet-LLH_glcm_MCC
	59	wavelet-HHH_glcm_JointEntropy
	60	wavelet-LLL_firstorder_Skewness
	61	wavelet-LLH_glrlm_ShortRunLowGrayLevelEmphasis
	62	wavelet-HLH_glrlm_LongRunEmphasis
	63	wavelet-LHL_gldm_GrayLevelVariance
	64	wavelet-HHL_glszm_GrayLevelNonUniformityNormalized
	65	wavelet-LLL_glszm_ZoneVariance
	66	lbp-3D-k_glszm_SizeZoneNonUniformityNormalized
	67	wavelet-LHH_glcm_JointEntropy
	68	original_glcm_Imc2
	69	lbp-3D-k_glcm_ClusterProminence

	70	original_glcm_SumEntropy
	71	wavelet-LLH_glcm_Id
	72	wavelet-HLH_glrlm_LowGrayLevelRunEmphasis
	73	wavelet-HLH_glszm_GrayLevelNonUniformityNormalized
	74	wavelet-LLH_glcm_Contrast
	75	wavelet-LHL_glszm_LowGrayLevelZoneEmphasis
	76	wavelet-LLH_glrlm_ShortRunHighGrayLevelEmphasis
	77	wavelet-HLH_firstorder_Minimum
	78	wavelet-HHH_glrlm_GrayLevelNonUniformityNormalized
	79	wavelet-HLL_firstorder_TotalEnergy
	80	wavelet-LHL_firstorder_Entropy
	81	wavelet-LLH_firstorder_Variance
	82	wavelet-LHH_firstorder_InterquartileRange
	83	wavelet-HLH_glrlm_LowGrayLevelRunEmphasis
	84	wavelet-LHH_glrlm_LongRunEmphasis
	85	wavelet-HLL_glrlm_RunPercentage
	86	wavelet-LLL_glszm_HighGrayLevelZoneEmphasis
	87	wavelet-LHL_glrlm_ShortRunHighGrayLevelEmphasis
	88	wavelet-HHH_gldm_DependenceVariance
	89	wavelet-LHL_glszm_HighGrayLevelZoneEmphasis
	90	wavelet-HHH_glrlm_GrayLevelVariance
	91	wavelet-HLH_glcm_ClusterShade
	92	wavelet-HLL_glszm_GrayLevelNonUniformityNormalized
	93	wavelet-LHL_glrlm_GrayLevelVariance
	94	wavelet-LLH_glcm_DifferenceEntropy
	95	wavelet-LLH_glcm_MCC
	96	wavelet-HHH_glcm_JointEntropy
	97	wavelet-LLH_glrlm_RunLengthNonUniformity
	98	wavelet-HHL_glszm_GrayLevelNonUniformityNormalized

	99	lbp-3D-k_glszm_SizeZoneNonUniformityNormalized
	100	wavelet-LHH_glcm_JointEntropy
	101	original_glcm_Imc2
	102	lbp-3D-k_glcm_ClusterProminence
	103	original_glcm_SumEntropy
	104	wavelet-LLH_glcm_Idn
	105	wavelet-HLH_glrlm_LowGrayLevelRunEmphasis
	106	wavelet-HLH_glszm_GrayLevelNonUniformityNormalized
	107	wavelet-LLH_glcm_Contrast
	108	wavelet-LHL_glszm_LowGrayLevelZoneEmphasis
	109	wavelet-LLH_glrlm_ShortRunHighGrayLevelEmphasis
